# Measurement and Modeling of Microbial Growth Using Timelapse Video

**DOI:** 10.3390/s20092545

**Published:** 2020-04-29

**Authors:** Konstantinos Delibasis, Ifigenia Basanou, Alexandros-Apostolos A. Boulogeorgos

**Affiliations:** 1 Department of Computer Science and Biomedical Informatics, University of Thessaly, 35100 Lamia, Greece; kdelimpasis@uth.gr (K.D.); ifigeniabasanou@hotmail.com (I.B.); 2 Department of Digital Systems, University of Piraeus, 18534 Piraeus, Greece

**Keywords:** image-based measurement, microbial growth model, timelapse video

## Abstract

The development of timelapse videos for the investigation of growing microbial colonies has gained increasing interest due to its low cost and complexity implementation. In the present study, a simple experimental setup is proposed for periodic snapshot acquisition of a petri dish cultivating a fungus of the genus Candida SPP, thus creating a timelapse video. A computational algorithm, based on image processing techniques is proposed for estimating the microbial population and for extracting the experimental population curves, showing the time evolution of the population of microbes at any region of the dish. Likewise, a novel mathematical population evolution modeling approach is reported, which is based on the logistic function (LF). Parameter estimation of the aforementioned model is described and visually assessed, in comparison with the conventional and widely-used LF method. The effect of the image analysis parameterization is also highlighted. Our experiments take into account different area sizes, i.e., the number of pixels in the neighborhood, to generate population curves and calculate the model parameters. Our results reveal that, as the size of the area increases, the curve becomes smoother, the signal-to-noise-ratio increases and the estimation of model parameters becomes more accurate.

## 1. Introduction

During the last decades, image capturing and processing have evolved from scarce technology, used by professionals for niche applications, to a rapidly advancing research field with application in low-cost and complexity data acquisition of long duration physical and/or biological phenomena [1]. To this end, a great amount of research effort has been spent on designing image processing and machine learning-based approaches that not only quantify the population growth for image sequences, but also provide accurate predictions concerning its evolution.

Scanning the open technical literature, there are several published papers that report the use of images and logistic functions (LFs) for predicting a specific-cell type population growth [2,3,4,5,6,7,8,9,10]. In more detail, in [2], the use of eight consecutive images, which were acquired by a single reflex (SLR) camera, was presented, in conjunction with the probability of survival of hamster stem cells, which was modeled according to the LF approach. Additionally, in [3], the authors showed that the in-vitro cellular growth rate may be used as survival probability of the fetus in case of hamsters, while, in [4], a multi-camera approach for modeling larva population by means of LF was presented. Another example of timelapse application in population behavior is delivered in [5], where the insect *Costelytra zealandica* was infected with the bacteria Yersinia entomophaga and its behavior was observed and recorded by acquiring 1 image per 10min, using a digital SLR camera. Fluorescence substances were used on the insects to facilitate analysis of the resulting image sequence. More recently, in [6], the construction of a special incubation chamber was described that uses two petri dishes to control humidity for a long duration. Images were acquired every 10min for 40 days and used to record and study the morphology of bacterial colonies, such as streptomycin, bacillus subtillis E42, serratia marcescens, arthrobacter agilis, and nestekonia SP. The timelapse technique has also been applied to agriculture [7], embryo moprhokinetics [8] and microbial interaction [9,10].

From the data processing point of view, a number of LF-based functions have been employed for modeling and/or predicting the population evolution based on the birth and death rates [11,12]. In this direction, in [13,14], the bi-LF was introduced to model population evolution that show two overlapping or sequential phases of logistic growth. The two LFs were summed and fitted to rice crop evolution data to handle an initial growth increase followed by a decrease. The combined curve provided a good fit to the data under investigation. Of note, non-linear least squares are used to estimate the parameter values of the function. Meanwhile, in [15], the LF is augmented by using an additive constant to accommodate varying capacity. This method was applied to two actual cases of human population growth. Moreover, in [16], the bi-phasic LF was used for modeling the sudden changes in the evolution of research papers and patents. The combination of two LFs was treated in a systematic way, using the following categories: (i) Sequential (sum of two time translated curves), (ii) superposed simultaneous curves, (iii) converging (first logistic growth combined with a second logistic function, reaching the maximum at about the same time) and (iv) diverging (different growth rates and carrying capacities). In all aforementioned cases, the resulting curves were unable to accommodate data decreasing trends. Additionally, in [17], a number of different extensions of the LF were discussed including the generalized logistic regression, the Von Bertalanffy, and the Blumberg expressions. Again, these expressions were unable to model decreasing phases of the data. Finally, in [18], the combertzian population growth model, which is a double exponential model, with parameters that control the time shift, the maximum asymptotic population and the maximum growth rate, was applied to investigate the evolution of breast cancer cells.

To the best of the authors’ knowledge, the problems of modeling the decreasing phase of the data have not been adequately addressed in the technical literature. Motivated by this, the current contribution presents an appropriate growth approach that is able to model both the increasing and decreasing phases and verifies its efficiency by means of experiment-driven microbial population modeling. In particular, we propose an experimental setup for periodic snapshot recording of the evolution of a fungus on a petri dish. The selected fungus is the Candida species, which is the most common cause of fungal infections [19,20,21,22]. The acquired images are processed in the red–green–blue (RGB) color system to quantify the number of microbes in each pixel of the plate. A novel mathematical model of the time evolution of the fungus for individual pixel/regions of the plate is presented, which introduces an LF with second degree polynomial time exponent capable of modeling population curves that may eventually become decreasing. Finally, we apply parametric imaging to depict the spatial distribution of a quantity proportional to the growth rate of the microbial population. The proposed approach is expected to open a new road to automatic data acquisition, modeling, and prediction of different evolution processes. The results are further validated and the robustness of the proposed method assessed using two more publicly available timelapse videos with a different acquisition method and microbial colonies of different species.

The rest of this paper is organized as follows: Section 2 is focused on presenting the methodology that we follow. In more detail, the experimental setups accompanied by the developed theoretical framework are reported in this section. Next, in Section 3 experimental results that verify the proposed analysis and the accuracy of the proposed modeling function are presented. Finally, useful remarks and conclusions summarizing this work are provided in Section 4.

Notations: Unless otherwise stated, in what follows, lower and upper case bold letters denote vectors and matrices, respectively. Moreover, A∪B stand for the union of the sets A and B, while ⊕ and •, respectively, represent the morphological dilation and erosion. Additionally, ${\left(\cdot \right)}^{T}$ and ${\left(\cdot \right)}^{-1}$, respectively, stand for the transpose and inverse operator. Finally, $\exp\left(\cdot \right)$ and $\ln\left(\cdot \right)$, respectively, denote the exponential and the natural logarithm.

## 2. Methodology

This section aims at reporting the methodology that is used for deriving and modeling the experimental data with the aid of LF with second degree (LFSD) exponent. In particular, in Section 2.1, the experimental setup is described, while the approach that we use to estimate the microbe population evolution and generate its curves is reported in Section 2.2. Finally, Section 2.3 presents the proposed time-shifted LFSD exponent approach, while the parametric imaging is reported in Section 2.4.

### 2.1. Experimental Setup

**Figure 1:**
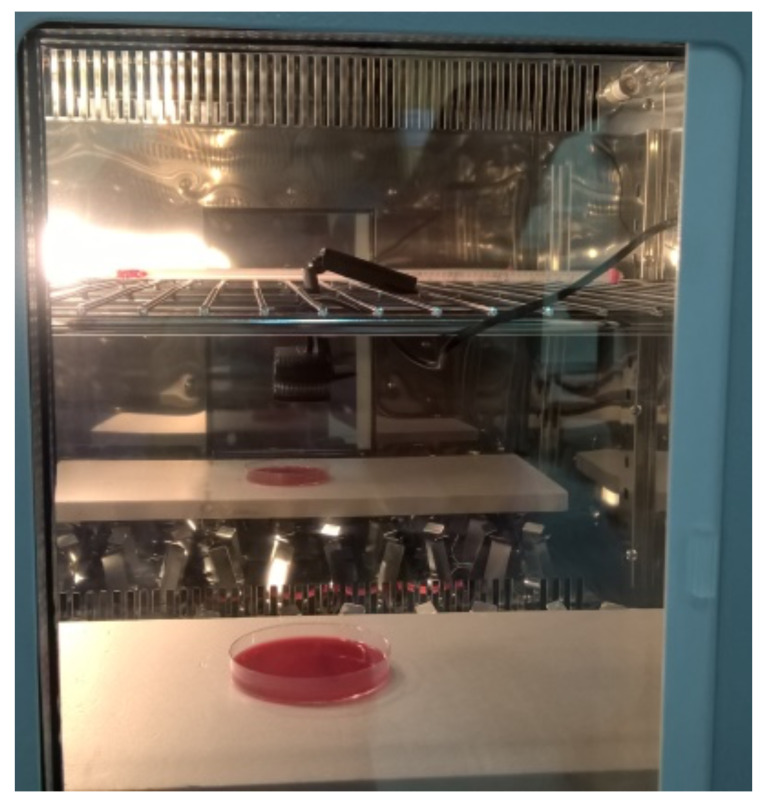
Experimental setup.

**Figure 2:**
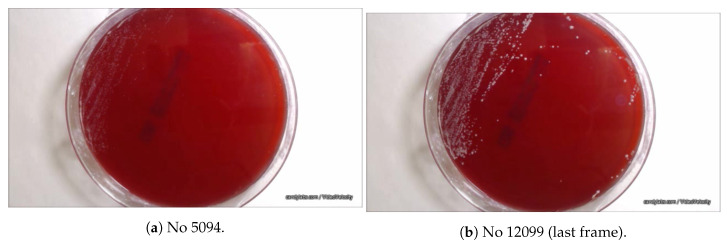
Two petri dish 1 frames in different microbial evolution states.

The experiment took place at the Department of Computer Science and Biomedical Informatics. The experimental setup consisted of a Logitec 920 camera, used to acquire images of the petri dish that was placed inside the incubator. As illustrated in Figure 1, an artificial light source of a white, 6Watt light emitting diode (LED) was used for illumination. Images were captured on an inexpensive laptop using the free version of video-velocity software (http://www.candylabs.com/videovelocity). The Candida SPP was used as a fungus. The evolution of Candida SPP took place on a Biomeriex cos petri dish. The red color of the plate is convenient for the post processing of the acquired images. The camera was placed directly above the petri dish, at an approximate distance of 15cm. Camera focus was set manually and motion detection was disactivated. Image resolution was set to 360×640 pixels. The period of frame acquisition was set to 15s and the fungus was allowed to grow for 50h, 24min and 36s, resulting in a total of 12,099 jpeg color images. The first 3800 of them contained no evidence of fungus evolution; thus, they were excluded from the study to facilitate further processing, leaving 8300 to participate in further processing. Indicative examples of the microbial evolution are presented in Figure 2.

In order to extent the validation of the proposed algorithm, as well as test its robustness, two more time lapse movies of microbial/fungi growth in petri dish were used, namely petri dish 2 and 3, respectively. In petri dish 2, a number of different cultures were recorded on a blood sheep of similar color as the one that we used in our experiment. The time lapse movie was obtained from Youtube (https://www.youtube.com/watch?v=XLmk0zYIFjE) and consists of 880 frames of 720 lines by 1280 columns. For petri dish 3, another timelapse movie was obtained as a publicly available file, described in [23]. The movie was acquired using a scanner, with a period of one frame every 5min. A total number of 2250 frames consisting of 720 lines by 1280 columns were extracted and processed. The frames were cropped inside the dish.

### 2.2. Image-Based Measurement of Microbe Population and Generation of Experimental Population Curves

As can be observed in Figure 2, the sheep blood agar plate 1 and 2 has a distinct red color. The fungal colonies color is different than the plate’s; thus, we make the fundamental assumption that the number of microbes of each imaged pixel is proportional to difference between the two colors. Formally, the number of microbes at each pixel (i,j) at time *t*, can be estimated by the difference of the red channel value $R_{i,j}^{t}$ from the corresponding value at the initial frame $R_{i,j}^{0}$. The total number of microbes are computed for all pixels in the current frame *t*.

The pixels that lie outside the plate should be excluded. In this direction, a binary mask, $M_{i,j}$, is defined with size equal to the one of the frame, which ideally should be 1 in all pixels inside the plate and 0 otherwise. In other words, the pixels inside the plate can be detected by using the following heuristic condition:

(1) $$\begin{array}{c} M_{i,j}=1\Leftrightarrow \left\{R_{i,j}^{0}\geq 2G\right\}\cap \left\{R_{i,j}^{0}\geq 2B_{i,j}^{0}\right\}, \end{array}$$

where $G_{i,j}^{0}$ and $B_{i,j}^{0}$ are, respectively, the values of the green and blue channel of pixel (i,j) in the initial frame. The binary mask in Equation (1) is further processed using the following morphological operation:

(2) $$\begin{array}{c} \tilde{M}=\left(M⊕B_{1}\right)•B_{2}, \end{array}$$

where M is a matrix with elements $M_{i,j}$, while $B_{1}$ and $B_{2}$ are, respectively, square structuring elements of size 11×11 and 31×31.

**Figure 3:**
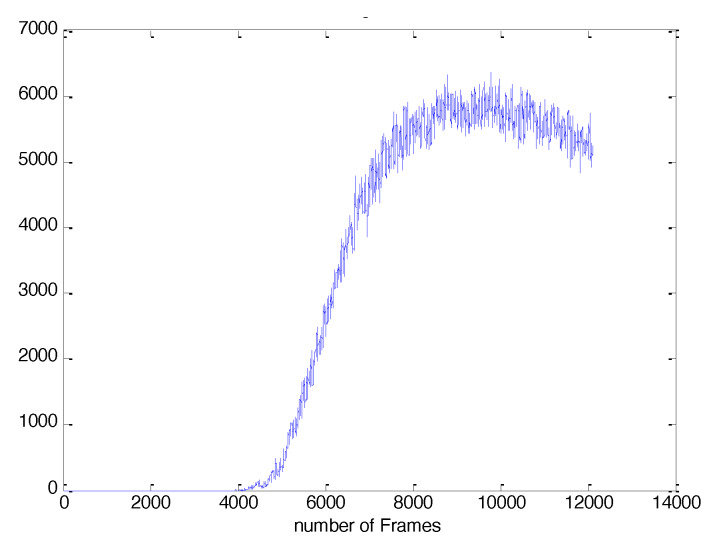
The evolution of total number of microbes in the whole plate, according to Equation (3).

The total number of microbes in the current frame *t* can be obtained as

(3) $$\begin{array}{c} N_{t}=\sum_{i,j}\Delta_{i,j}^{t}{\tilde{M}}_{i,j}, \end{array}$$

where ${\tilde{M}}_{i,j}$ is the i,j element of the matrix $\tilde{M}$, while

(4) $$\begin{array}{c} \Delta_{i,j}^{t}=\left\{\begin{array}{cc} R_{i,j}^{t}-R_{i,j}^{0}, & \left|R_{i,j}^{t}-R_{i,j}^{0}\right|\geq T\\ 0, & \mathrm{otherwise} \end{array}\right., \end{array}$$

with *T* being a threshold, which is applied in order to prevent insignificant color changes from contributing to the population calculation. The threshold is experimentally determined and is set to 10. The total estimated number of microbes in the whole plate for each frame is illustrated in Figure 3.

In the case of the third petri dish, which is radically different than the red-colored circular petri dishes 1 and 2, the following minor modifications were made to the image-based measurement algorithm. Since the whole frame contains microbial colonies there is no need to define the binary mask as in Equations (1)–(3) can be simplified as $M_{ij}=1$ one for all i,j. Finally, since the original color of the plate is black, Equation (4) should be modified, in order to take into account all chromatic channels, as

(5) $$\begin{array}{c} \Delta_{ij}=\max\left(r_{ij},g_{ij},b_{ij}\right), \end{array}$$

where

(6) $$\begin{array}{cc} r_{ij} & =\left\{\begin{array}{cc} |R_{ij}^{t}-R_{ij}^{0}|, & \mathrm{for}\;|R_{ij}^{t}-R_{ij}^{0}|\geq T\\ 0, & \mathrm{otherwise} \end{array}\right., \end{array}$$

(7) $$\begin{array}{cc} g_{ij} & =\left\{\begin{array}{cc} |G_{ij}^{t}-G_{ij}^{0}|, & \mathrm{for}\;|G_{ij}^{t}-G_{ij}^{0}|\geq T\\ 0, & \mathrm{otherwise} \end{array}\right., \end{array}$$

and

(8) $$\begin{array}{cc} b_{ij} & =\left\{\begin{array}{cc} |B_{ij}^{t}-B_{ij}^{0}|, & \mathrm{for}\;|B_{ij}^{t}-B_{ij}^{0}|\geq T\\ 0, & \mathrm{otherwise} \end{array}\right.. \end{array}$$

From Equation (3), it becomes evident that the population of microbes in a region of interest (ROI), *A*, of the plate can be evaluated as

(9) $$\begin{array}{c} N_{t}\propto \sum_{\left(i,j\right)\in A}\Delta_{i,j}. \end{array}$$

**Figure 4:**
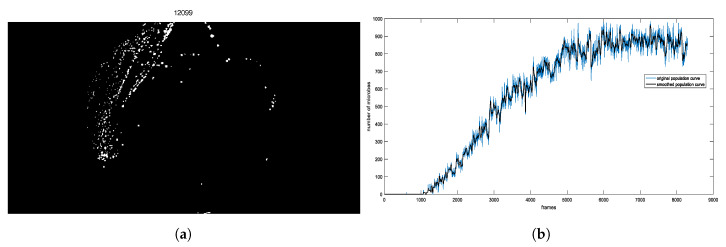
(**a**) The estimated number of microbes according to Equation (9) for each pixel, encoded in shades of gray. (**b**) A curve example and its smoothing effect at a random point of plate by using median filter.

The ROI is a square defined round its central pixel of size $\left(2r+1\right)\times \left(2r+1\right)$. An indicative example is presented in Figure 4a, where the result of applying Expression (9) to each pixel of the last frame for r=8 pixel is provided. Figure 4b depicts a typical example of an experimental population curve (blue) accompanied by the smoothed population one (black). The experimental curves show statistical fluctuations, due to inaccuracies of the estimation method of the population, which are considered as noise. In order to increase the accuracy of population modeling function, smoothing of the extracted curve is necessary. In this direction, we employ a 27-element median filter.

The instantaneous signal-to-noise ratio (SNR) is defined as

(10) $$\begin{array}{c} \gamma \left(t\right)=\frac{s(t)}{n(t)}, \end{array}$$

where s(t) and n(t) are, respectively, the signal and noise amplitudes. Note that the signal amplitude is measured as the mean value of the curve, while the noise amplitude is calculated as the standard deviation of the curve at the same time interval. Let us consider $t_{p}$ points before and after the current frame, *t*, then s(t) and n(t) can be, respectively, obtained as

(11) $$\begin{array}{cc} s(t) & =\frac{1}{2t_{p}+1}\sum_{\tau =t-t_{p}}^{t+t_{p}}N_{\tau }, \end{array}$$

(12) $$\begin{array}{cc} n(t) & =\sqrt{\frac{1}{2t_{p}+1}\sum_{\tau =t-t_{p}}^{t+t_{p}}\left(N_{\tau }-s\left(\tau \right)\right)}. \end{array}$$

### 2.3. The Proposed Time-Shifted LFSD Exponent

At the onset of the LF model, the population grows exponentially, but after some time, the rate of increase reduces until it reaches zero. Thus, the LF approximates asymptotically a fixed value that represents a theoretical maximum limit to the population size under given conditions, such as the resource availability decrease, toxic products concentration, etc. Let $p_{\mathrm{LF}}\left(t\right)$ denote the percentage of the population maximum at time *t*, which can be expressed as

(13) $$\begin{array}{c} p_{\mathrm{LF}}\left(t\right)=\frac{1}{1+\exp\left(-\left(b_{1}t+b_{0}\right)\right)}, \end{array}$$

where $b_{0}$ and $b_{1}$ are LF-specific parameters.

As the colony evolves further, it is possible for its actual population curve to exhibit a downward trend and become decreasing, due to lack of nutrients, or the accumulation of toxic metabolic products. In order to model this type of population trend, we modify the conventional LF by introducing a second degree polynomial as exponent. The LFSD can be written as

(14) $$\begin{array}{c} p_{\mathrm{LFSD}}\left(t\right)=\frac{1}{1+\exp\left(-\left(b_{2}t^{2}+b_{1}t+b_{0}\right)\right)}. \end{array}$$

In contrary to the conventional LF, the LFSD cannot be easily adjusted to fit time-translated experimental data. That would require the exponent of LFSD to become $-\left(b_{2}{\left(t-t_{s}\right)}^{2}+b_{1}\left(t-t_{s}\right)+b_{0}\right)$, introducing an extra parameter $t_{s}$, which cannot be determined by solving a linear system of equations. Instead, we alleviate this problem by measuring experimentally the time $t_{s}$ of the first appearance of non-zero experimental data. Thus, given the values of the population $N_{i}$, for i=1,⋯,n, where *i* represents different time values, LFSD can be rewritten as

(15) $$\begin{array}{c} p_{\mathrm{LFSD}}\left(t+t_{s}\right)=\frac{1}{1+\exp\left(-\left(b_{2}t^{2}+b_{1}t+b_{0}\right)\right)}, \end{array}$$

where $b_{0}$, $b_{1}$, and $b_{2}$ are LFSD-specific parameters. In order to use Equation (15) for modeling the experimental population curve of a colony on the plate, we have to normalize the population curve in the range $\left[0,1\right]$. In this direction, we set

(16) $$\begin{array}{c} p_{t}=\frac{N_{t}}{N_{\max}+e}, \end{array}$$

where $N_{\max}$ is the maximum value of the population curve and *e* is a small positive number.

From Equation (15), we can obtain a linear form that facilitates the calculation of parameter $b_{0}$, $b_{1}$, and $b_{2}$, as

(17) $$\begin{array}{c} y_{i}=\ln\left(\frac{p_{\mathrm{LFSD}}\left(t_{i}+t_{s}\right)}{1-p_{\mathrm{LFSD}}\left(t_{i}+t_{s}\right)}\right), \end{array}$$

or equivalently

(18) $$\begin{array}{c} y_{i}=b_{2}t_{i}^{2}+b_{1}t_{i}+b_{0}. \end{array}$$

Given the values of $\left(t_{i},y_{i}\right)$ with i=1,⋯,N, the parameters $b_{0}$, $b_{1}$, and $b_{2}$ can be obtained as the solution of

(19) $$\begin{array}{c} Tb=y, \end{array}$$

where $b={\left[b_{1},b_{2},b_{3}\right]}^{T}$, $y=\left[y_{1},\cdots ,y_{N}\right]$, and

(20) $$\begin{array}{c} T=\left[\begin{array}{ccc} t_{1}^{2} & t_{1} & 1\\ \vdots & \vdots & \vdots \\ t_{N}^{2} & t_{N} & 1 \end{array}\right]. \end{array}$$

From Equation (19), b can be calculated as

(21) $$\begin{array}{c} b={\left(T^{T}T\right)}^{-1}T^{T}y. \end{array}$$

**Theorem** **1.** *The maximum value of the population curve can be achieved at*

(22) $$\begin{array}{c} t_{\max}=-\frac{b_{1}}{2b_{2}}+t_{s}. \end{array}$$

**Proof.**  The growth rate can be evaluated as

(23) $$\begin{array}{c} \rho =\frac{dy_{i}}{dt_{i}}, \end{array}$$

or equivalently

(24) $$\begin{array}{c} \rho =\frac{dy_{i}}{dp_{LFSD}\left(t_{i}+t_{s}\right)}\frac{dp_{LFSD}\left(t_{i}+t_{s}\right)}{dt_{i}}, \end{array}$$

which can be analytically written as

(25) $$\begin{array}{c} \rho =C\left(2b_{2}t_{i}+b_{1}\right)\exp\left(-\left(b_{2}t_{i}^{2}+b_{1}t_{i}+b_{0}\right)\right), \end{array}$$

where

(26) $$\begin{array}{c} C=\frac{1}{p_{LFSD}\left(t_{i}+t_{s}\right)\left(1-p_{LFSD}\left(t_{i}+t_{s}\right)\right)}. \end{array}$$

In order to evaluate $t_{\max}$, we set ρ=0 and we get Equation (22). □

**Remark** **1.** *Note that if the population curve is monotonically increasing during the time period of image acquisition, then Equation (22) returns a value outside the time frame. This value should be discarded.*

**Figure 5:**
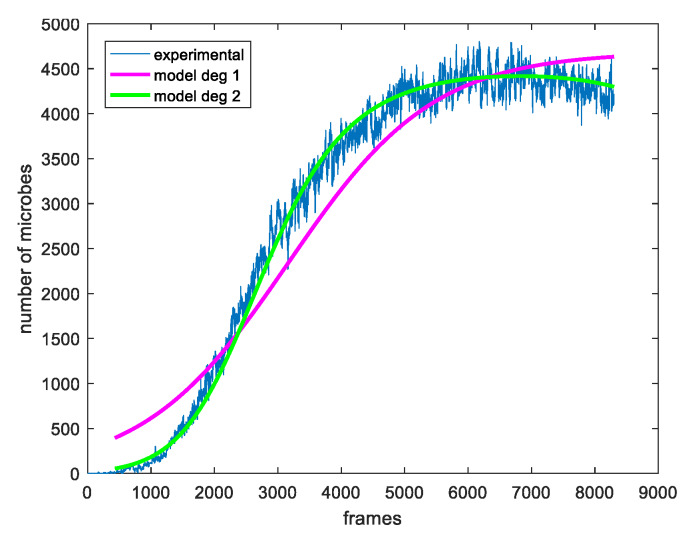
The experimental curve of the fungi on the whole plate is illustrated in blue and the modeling of the logistic function (LF) with linear and square exponent with pink and green, respectively.

An example of determining the model parameters for the experimental population curve of the plate that exhibits a late decreasing phase is shown in Figure 5. Modeling the experimental data using LF and LFSD is performed, respectively shown in pink and green curve. From this figure, it becomes evident that the LFSD achieves more accurate modeling compared to the LF, because the former is able to model the last phase, when the population declines, while, in the deceasing phase, the latter increases monotonically with its slope approaching zero.

### 2.4. The Parametric Image

**Figure 6:**
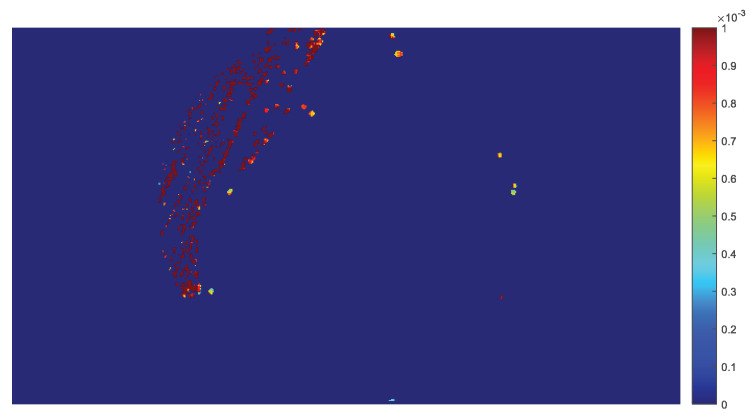
The parametric image resulting from the determination of the linear exponent $b_{2}$ of the LF with second degree (LFSD), for each non-zero pixel of the image in the final frame, using the region of interest (ROI) with r=4 pixels.

It is possible to apply the experimental population curve extraction and its parameter estimation to each pixel of the frame, so that the values of the model parameters can be calculated separately for each non-zero pixel and displayed as a parametric image. In this study, parametric images are constructed for the parameters $b_{2}$ and $b_{1}$ of the LFSD, $b_{1}$ of the LF, $t_{\max}$ as calculated by Equation (22) from the LFSD and $t_{0}$. Therefore, the parametric image shows regions of the plate with a different population growth rate. Figure 6 shows the value of the coefficient $b_{2}$ for the LFSD model for ROI with r=4.

## 3. Results

**Figure 7:**
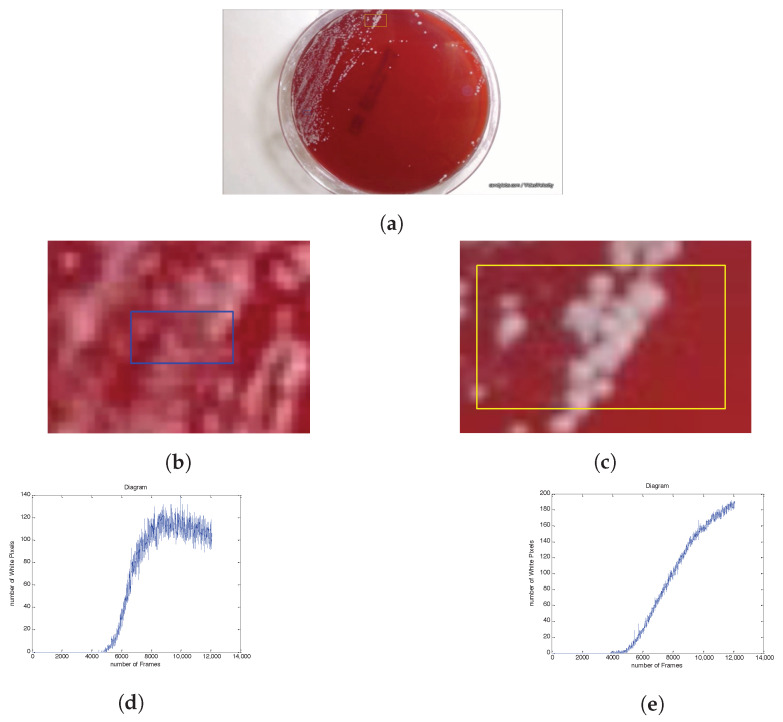
(**a**) The last snapshot of the petri dish, along with two selected areas containing many colonies, depicted in yellow and blue; (**b**,**c**) the selected areas of the plate magnified; (**d**,**e**) the corresponding experimental population curves of the blue and yellow areas, respectively.

This section is devoted to presenting experimental results that evaluate the effectiveness and accuracy of the proposed approach. In this direction, in Figure 7, an indicative example is depicted, where, in the final acquired frame, two regions of the plate, which contain several colonies, are selected. The extracted population curves are also presented in Figure 7d,e. It is evident that the curve of the smaller blue area, which is depicted in Figure 7b,d, shows a decreasing trend. Finally, as illustrated in Figure 7c,e, the evolution curve of the yellow area has a monotonically increasing trend.

**Figure 8:**
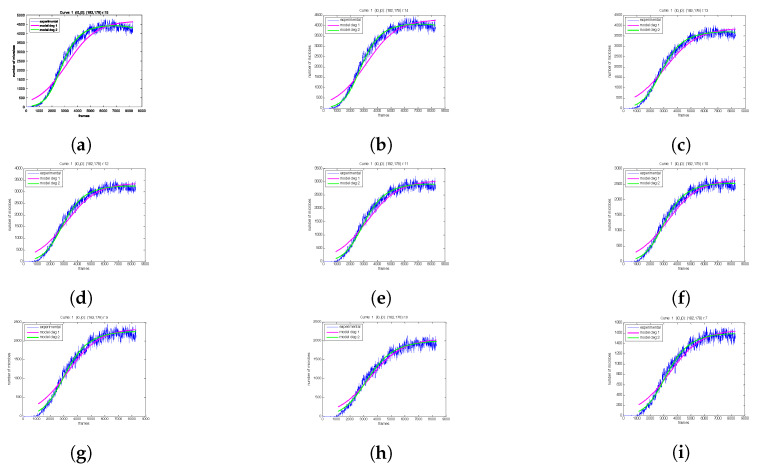
The modeling of the generated experimental curves with LF and LFSD for a different size area *r*, around the central point. (**a**) *r* = 15, (**b**) *r* = 14, (**c**) *r* = 13, (**d**) *r* = 12, (**e**) *r* = 11, (**f**) *r* = 10, (**g**) *r* = 9, (**h**) *r* = 8, (**i**) *r* = 7.

Next, the influence of the selected area size is explored. First, a pixel is selected and the experimental population curve is calculated for different sizes of the square region centered at the selected pixel. As depicted in Figure 8, for each experimental curve, the modeling is performed using both the LF and LFSD approaches. Moreover, the LFSD approach is observed to be more accurate compared to the LF. Interestingly, it becomes obvios that as the value of *r* decreases, the population curve becomes more noisy.

**Figure 9:**
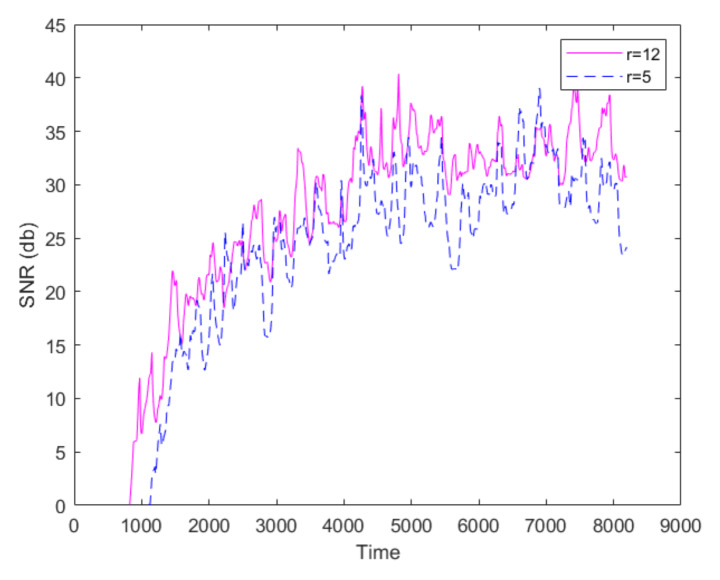
The instantaneous signal-to-noise ratio (SNR) for the population curve in Figure 8, calculated for ROI size r=5 and r=12.

For each *r* parameter, the coefficients of the exponential polynomial in the case of LF and LFSD are, respectively, reported in Table 1 and Table 2. Notice that $b_{1}$ in the case of LF and $b_{2}$, $b_{1}$ of LFSD are population growth rate metrics. The consistency of the curve extraction and proposed modeling is shown in Figure 9, where $t_{\max}$ is calculated using Equation (22). It can be observed that $t_{\max}$ does not vary substantially even for drastically *r* changes.

**Figure 10:**
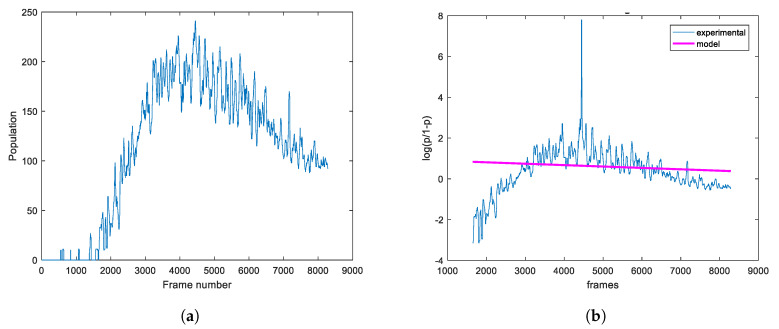
(**a**) An example of a population curve with negative *b*_1_ of the LF. (**b**) The logarithmic quotient and its classic LF modeling.

**Figure 11:**
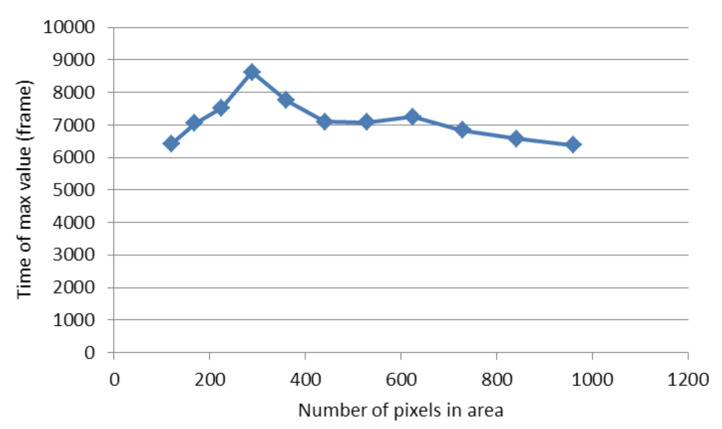
The value of $b_{1}$ coefficient of the LF as a function of the number of pixels in ROI.

To highlight the superiority of LFSD against the conventional LF, in Figure 10a, we present a population evolution, which after a specific frame is decreasing. Figure 10b illustrates the estimated population against the LF-based model. From this figure, it becomes evident that the conventional LF is incapable of modeling this behavior. This highlights the importance of LFSD for modeling such trends. The number of pixels with negative $b_{1}$ in the conventional LF in the whole petri dish is also reported in Table 3. We notice that the number of pixels having negative exponent $b_{1}$ decreases as the radius of the ROI area increases (Figure 11). However, the proposed LFSD does not suffer from this shortcoming.

The extracted population curves exhibit noticeable fluctuation, which appears to increase as the local mean value of the curve increases. Figure 9 plots the instantaneous signal-to-noise ratio (SNR) for the population curve shown in Figure 8. The parameter $t_{p}$ is set to 80. The logarithmic definition of SNR was used. It becomes apparent that as the population increases with time, both the amplitude of signal and noise increases, however the signal amplitude increases faster than the noise (curve’s local standard deviation); thus, the SNR overall increases. Furthermore, as expected, the noise decreases as the size of the ROI used for the calculation increases.

**Figure 12:**
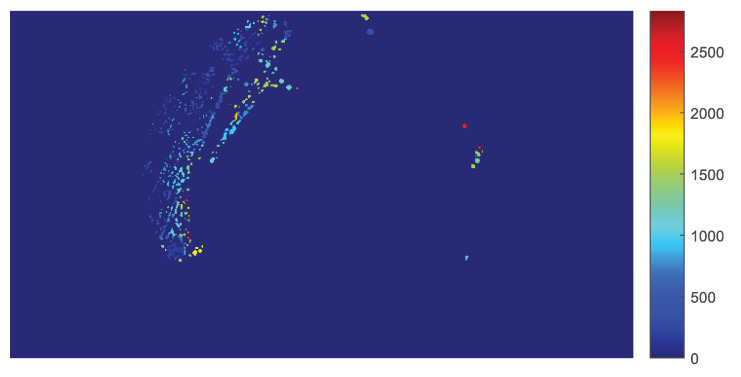
Parametric image of the time of first microbial appearance $t_{s}$.

The time $t_{s}$ of the first appearance of non-zero experimental data for each pixel is shown as a parametric image in Figure 12.

**Figure 13:**
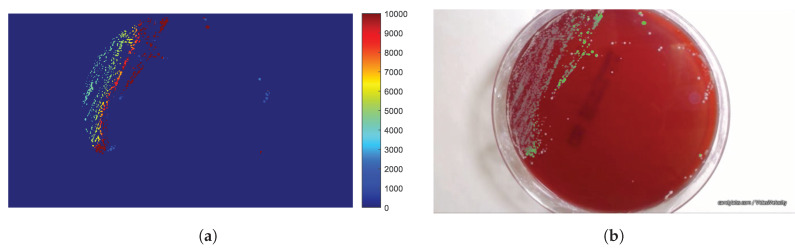
The pixels with observed zero growth rate during the experiment, shown in green, superimposed on the final frame of the petri dish.

Similarly, $t_{\max}$ is shown in Figure 13a and also superimposed on the petri dish in Figure 13b, using shades of green.

**Figure 14:**
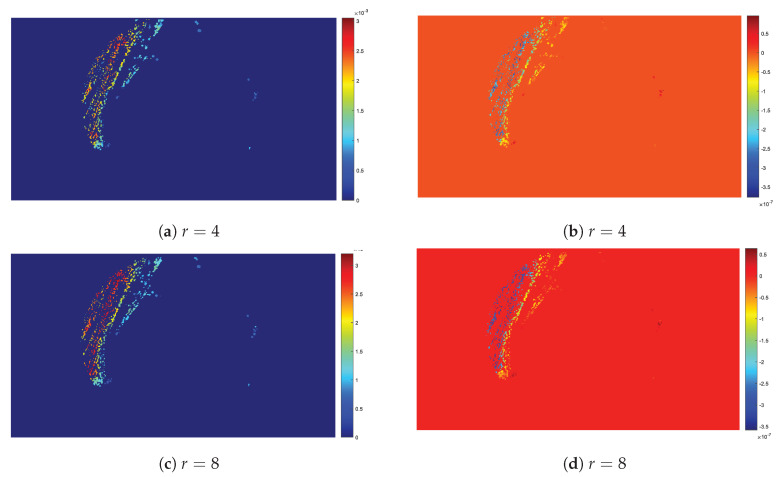
The effect of *r* to the determination of *b*_2_ (**a**,**c**) and *b*_1_ (**b**,**d**) for the LFSD population model are shown for *r* = 4, 8.

Finally, Figure 14 shows parametric images of $b_{2}$ of LF, with their color scale, for two different radii r=4 and 8 pixels, respectively. Visual inspection of the parametric images $b_{1}$ and considering the influence of the $b_{1}$ factor on the population curve, suggests that areas with a high parameter values exhibit a low population density.

**Figure 15:**
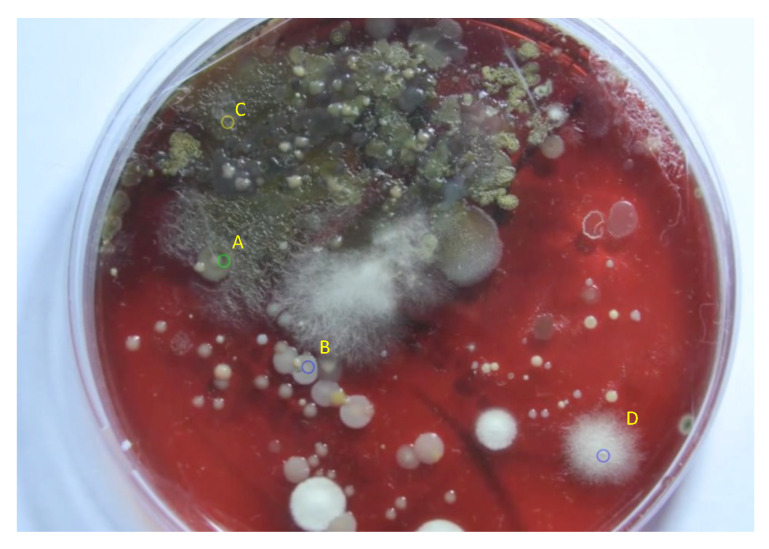
The final frame of petri dish 2, with four locations marked on different colonies, from which population curves are extracted and modeled.

**Figure 16:**
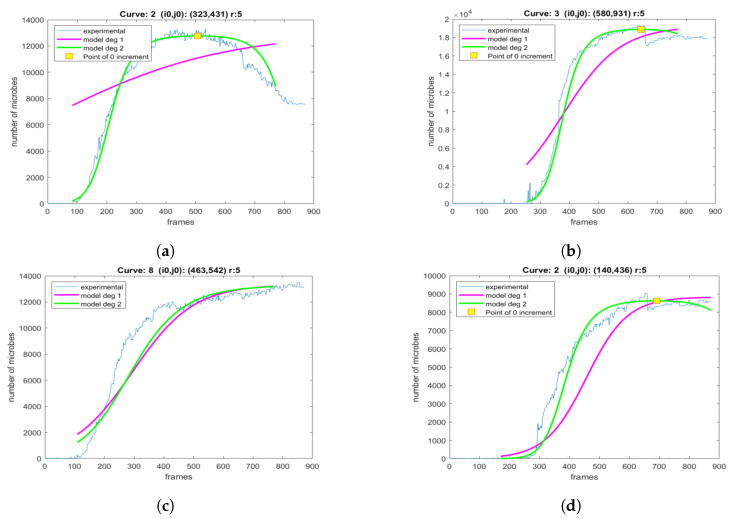
The modeling of the generated experimental curves with LF and LFSD for area of size *r* = 5, around the central point. The curves are extracted from locations marked as (**a**) A, (**b**) B, (**c**) C, and (**d**) D on the final frame of the petri dish 2.

In Figure 15, four locations are shown on different colonies of petri dish 2 in the final frame. The population curves that were extracted and modeled using the LF and the proposed LFSD, are shown in Figure 16. Again, it can be observed that the LFSD achieves more accurate population modeling in the cases of populations reaching their maximum during the acquisition, as well as in the case of population’s continuous grow.

**Figure 17:**
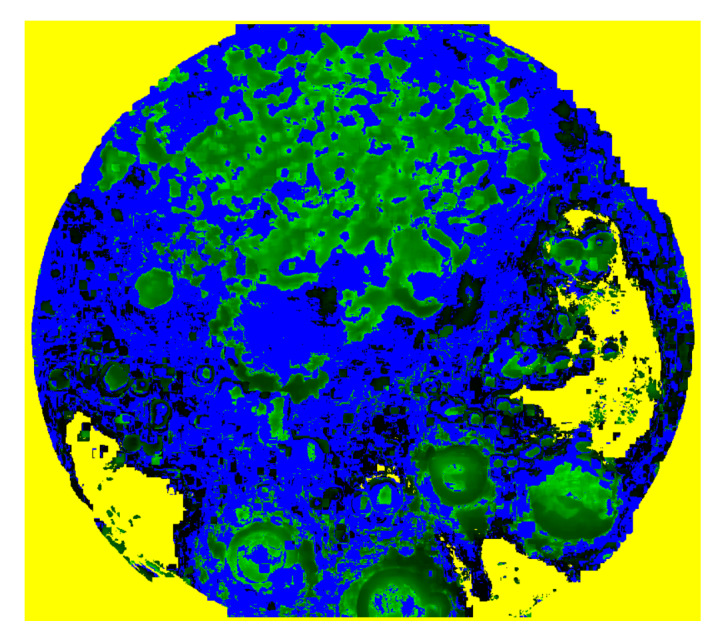
The final frame of petri dish 2, with four locations marked on different colonies, from which population curves are extracted and modeled.

The parametric image of petri dish 2 that depicts the time in which the population is maximized, is shown in Figure 17. Blue color represents pixels with continuously increasing population that did not exhibit maximum value during the acquisition, yellow color marks pixels whose $t_{\max}$ cannot be determined because the curve is too noisy to be modeled (or pixels outside the dish) and finally shades of green color (from black to light green) encode the determined value of $t_{max}$ for the remaining pixels, in the range [0,900] frame. Note that even the yellow pixels inside the petri dish appear in areas where no visible colony growth could be detected

**Figure 18:**
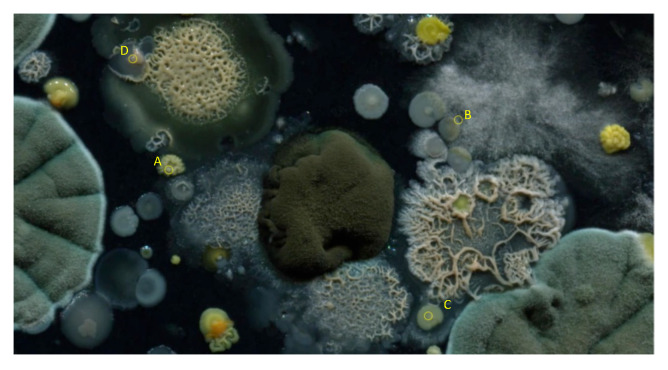
The final frame of petri dish 3, with four locations marked on different colonies.

**Figure 19:**
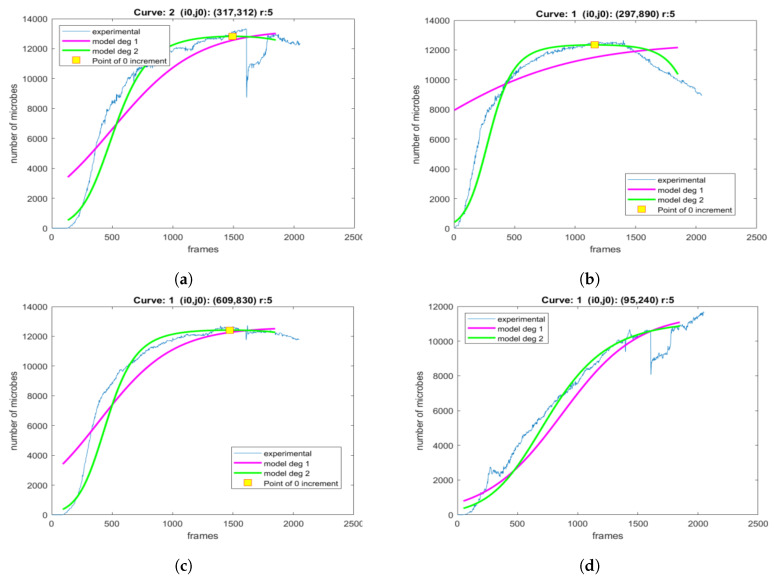
The modeling of the generated experimental curves with LF and LFSD for area of size *r* = 5, around the central point. The curves are extracted from locations marked as (**a**) A, (**b**) B, (**c**) C, and (**d**) D on the final frame of the petri dish 2.

In Figure 18, four locations are shown on different colonies of petri dish 3. The extracted population curves and the extracted LF and LFSD-based models are depicted in Figure 19. It can be observed that the LFSD achieves more accurate population modeling.

## 4. Conclusions

In this paper, a low-complexity approach for petri dish image acquisition, automatic measure and mathematically modeling fungus evolution was presented. The images were acquired using an inexpensive web camera. An image-based heuristic method for microbial number estimation was described and applied for the generation of experimental population curves. A novel modeling approach based on the LFSD was presented for the population evolution and compared with the conventional LF. Our results highlighted that the the proposed approach outperforms the commonly-used LF one, in terms of accuracy, since it can also capture decreasing trends of the population evolution. The concept of parametric imaging was studied in order to further improve the visualization of the LFSD model parameters. Finally, the different origins of the three petri dishes, the variety of image acquisition process, image quality and resolution and the different species of growing microorganisms, show the robustness of the proposed population measurement and modeling method.

Future work may include comparison between the growth measures identified in this work, such as the rate of growth and the time of maximum population, between different cultures of the same or different micro-organisms. Such comparisons also require identical image acquisition conditions and equipment and possibly color histogram normalization of the images, adapted to the background color of each individual dish.

## Figures and Tables

**Table 1:** The values of $b_{1}$ of LF for different values of *r*, for the selected pixel of the dish.

Size of Are (Pixel)	Number of Pixels in the Area	$b_{1}\left(\times 10^{-3}\right)$	$b_{0}$
7	225	8.39	−1.918
8	289	8.44	−1.962
9	361	8.46	−1.811
10	441	8.61	−2.040
11	529	8.51	−1.976
12	625	8.68	−2.004
13	729	8.20	−1.828
14	841	8.68	−2.263
15	961	8.72	−2.386

**Table 2:** The values of $b_{2}$, $b_{1}$, $b_{0}$ of LFSD for different values of *r*, for the selected pixel of the dish.

Size of Are (Pixel)	Number of Pixels in the Area	$b_{2}\left(\times 10^{-7}\right)$	$b_{1}\left(\times 10^{-3}\right)$	$b_{0}$
7	225	−1.06	1.59	−2.934
8	289	−0.78	1.35	−2.647
9	361	−1.01	1.57	−2.786
10	441	−1.26	1.78	−3.316
11	529	−1.25	1.77	−3.246
12	625	−1.23	1.78	−3.273
13	729	−1.31	1.79	−3.182
14	841	−1.60	2.11	−4.052
15	961	−1.78	2.27	−4.431

**Table 3:** The effect of *r* to the determination of $b_{1}$ for the conventional LF population model.

*r*	Number of Pixels with $b_{1}<0$
2	376
4	229
8	85
